# Beyond representations: from active inference to active interference

**DOI:** 10.3389/fnhum.2026.1925538

**Published:** 2026-09-15

**Authors:** Tommaso Firaux, Natasha Greenstein, Jack Tuszynski, Marco Cavaglià

**Affiliations:** 1 Department of Mechanical and Aerospace Engineering, Polytechnic University of Turin, Turin, Italy; 2 Center for Brain and Cognition, Universitat Pompeu Fabra, Barcelona, Spain

**Keywords:** active inference, active interference, Markov blanket, membrane biophysical models, predictive processing, lipid membrane, holographic encoding, biological autonomy

## Abstract

Active inference describes the system–environment boundary through statistical constructs such as the Markov blanket, a substrate-independent formalism that specifies a boundary's functional role without committing to the physical structure realizing it. We ask what structure can perform that role, and propose Active Interference as a complementary, biophysically grounded layer beneath the statistical description. We argue that biological boundaries are physically instantiated, resonant interfaces—lipid membranes coupled to vicinal water and endogenous electromagnetic fields—capable of wave superposition and history-dependent feedback. We present a minimal formalism for interference-based write/read cycles in which the substrate does not carry a representation but constitutes it. Inference is thereby grounded in interference, yielding an ontology of biological autonomy with phase-specific, falsifiable predictions.

## 1 Introduction: from representations to physical interfaces

Contemporary theories increasingly frame perception, action, and learning in representational and inferential terms (von Helmholtz, 1867; Gregory, 1980; Dayan et al., 1995; Rao and Ballard, 1999; Knill and Pouget, 2004; Clark, 2013, 2015; Hohwy, 2013): biological agents build internal models of the world and update them through sensory evidence. The most influential formulation, active inference, describes cognition as the minimization of variational free energy by updating beliefs about the hidden causes of sensory inputs (Friston, 2010, 2013; Friston et al., 2017), with the agent–environment boundary formalized through Markov blankets, statistical constructs partitioning internal from external states via conditional dependencies (Pearl, 1988; Friston, 2019; Kirchhoff et al., 2018). The formalism is powerful, offering a mathematically coherent account of adaptive behavior across scales, from cellular processes and morphogenesis to interoceptive regulation and whole-brain dynamics (Kirchhoff et al., 2018; Parr et al., 2022; Levin, 2024; Barrett and Simmons, 2015; Palacios et al., 2020; Sajid et al., 2021), and applying equally to subcellular signaling and morphogenesis (Levin, 2024; Friston et al., 2015; Pio-Lopez et al., 2022; Fields et al., 2024), synaptic plasticity (Friston et al., 2017; Bogacz, 2017), allostatic control (Barrett and Simmons, 2015; Seth and Friston, 2016; Barrett, 2017), affective states (Seth, 2013), plant behavior (Calvo and Friston, 2017), immunological inference (Bhat et al., 2021), social cognition (Ramstead et al., 2018; Veissière et al., 2020), and autonomous agent design (Lanillos et al., 2021). This generality derives from the construction itself: any system describable by coupled stochastic differential equations with a non-equilibrium steady-state density admits, under certain conditions, a Markov blanket partition (Friston, 2019).

Yet, the blanket is fundamentally a statistical property of probabilistic graphical models, not a physical boundary. It captures conditional independence between variables but says nothing about the material mechanisms through which a system exchanges energy, matter, and signals with its environment, and its existence depends on the observer's choice of partition rather than an intrinsic physical structure (Biehl et al., 2021; Bruineberg et al., 2018). The blanket is non-unique, admitting infinitely many partitions depending on how the state space is carved (Biehl et al., 2021); it is scale-dependent in an ontologically ambiguous way, since a blanket drawn around virtually anything cannot distinguish a living cell from an eddy or a candle flame (Bruineberg et al., 2018); and, most critically, it is statistical, not physical. Friston (2019) himself treats it as a property of the formalism rather than an ontological claim. Critics have pressed the point: Biehl et al. (2021) showed the mapping from steady-state densities to blanket structures is many-to-one; Raja et al. (2021) called explaining cognition via Markov blankets a sleight of hand, a modeling tool treated as an explanatory mechanism; and Bruineberg et al. (2018) held that the predictive power of the free energy principle (FEP) is limited by its lack of a material basis for its boundaries.

A Markov blanket specifies that a separation exists; it does not specify what holds it in place. The substrate the formalism posits is statistical—a partition of variables—and a partition does not generate the dynamics it describes, it presupposes them. What the account lacks is a physical boundary: a structure that does the separating.

None of this refutes the FEP; it shows that a complete account of autonomy must specify the physical mechanisms through which boundaries are implemented and maintained. That demand is not peculiar to the neural case, and it has already been met at the molecular scale: within Chemical Artificial Intelligence, single photochromic compounds, widespread across all kingdoms of life, are read as physical instantiations of a blanket, with the uncolored form as sensor, the photoproduct as actuator, and the manifold of excited states populated on absorption as internal states (Gentili, 2025). We therefore place physical boundaries at the center of biological organization. Building on the Unified Holographic Framework (UHF) for neural computation and consciousness Cavaglià and Tuszynski, (2025a) and its scale-free extension, the Energy–Mass–Information (EMI) model (Firaux et al., 2025), we argue that autonomy emerges not from statistical separation alone but from active interfaces: physically instantiated boundaries that mediate non-linear feedback between system and environment. Lipid membranes, the only universal boundary shared by all living organisms from bacteria to neurons, are the natural candidate, behaving as excitable, resonant media that reflect, transmit, and modulate oscillatory activity (Heimburg and Jackson, 2005; Shrivastava et al., 2015; Cavaglià et al., 2023). Cognition is thus reframed not as probabilistic inference over internal representations but as the emergence of structured dynamics at these interfaces, a transition from active inference to active interference. This rests on concrete grounds: Cavaglià and Tuszynski (2025a) propose that action potentials may supply enough energy to drive weak Fröhlich-type collective oscillations in phospholipid dipoles, generating coherent electromagnetic activity at the membrane interface, and complementary work shows that interference-based computation in lipid interfaces is physically realizable (Heimburg and Jackson, 2005; Shrivastava, 2024). The membrane is not a passive barrier that happens to implement a Markov blanket but an active medium whose non-linear, excitable, resonant properties underwrite the dynamical coupling between system and environment.

The paper proceeds as follows. Section 1 examines the limits of Markov blankets as biological boundaries; Section 2 introduces lipid membranes as active interfaces; Section 3 articulates the transition to active interference with a formal framework; Section 4 develops information-in-action through holographic encoding; Section 5 presents converging validation from biophysics and engineering; Section 6 discusses implications for neuroscience and biological autonomy; Section 7 concludes.

## 2 Biological boundaries as active interfaces

Following the Unified Holographic Framework (UHF) and its Energy–Mass–Information (EMI) paradigm (Cavaglià and Tuszynski, 2025a; Firaux et al., 2025), we describe a multilayered substrate in which the membrane operates not as a passive barrier but as a non-linear, excitable, phase-sensitive medium supporting wave propagation, interference, and distributed information encoding. The framework rests on four postulates—the membrane–water interface as a holographic plate (1), structured water as an effective coherence medium (2), read/write cycles via electromagnetic (EM)–hydration coupling (3), and multiscale boundary conditions set by the cerebrospinal-fluid cavity (4)—of which the last three are drawn on directly below; the full statements are given in (Cavaglià and Tuszynski 2025a). The biophysics is developed below; its implications for biological inference are taken up in Section 6.

The membrane–water system constitutes a complete active interface, combining six physical properties that no statistical partition specifies (Figure 1):

(i) **Nonlinear excitability**. Far from the passive bilayer of the fluid mosaic model (Singer and Nicolson, 1972), biological membranes display broad chain-melting transitions reaching physiological temperatures, shiftable by membrane tension, pH, ionic strength, and anesthetics (Cavaglià and Tuszynski, 2025a; Cavaglià et al., 2023; Heimburg, 2023; Ebel et al., 2001). Near the transition the membrane becomes excitable (Heimburg, 2023), with lateral compressibility rising by orders of magnitude alongside anomalous permeability and fluctuation-induced channel-like conductance events.

(ii) **Solitonic wave propagation**. We treat the action potential as a coupled electromechanical perturbation that reorganizes headgroup dipole order and generates the phase-structured excitations ψ(*x, t*) of Equation 1 (Cavaglià and Tuszynski, 2025a), converging with Heimburg and Jackson's soliton theory (Heimburg and Jackson, 2005), in which a density pulse propagates by transiently driving the bilayer through its chain-melting transition, accounting for the mechanical, thermal, and optical signatures of nerve impulses left unexplained by Hodgkin–Huxley. The same framework yields a unified account of anesthesia via the Meyer–Overton correlation (Heimburg and Jackson, 2007); in our terms, anesthetics disrupt dipolar coherence by altering membrane mechanics (Zizzi et al., 2022) and reducing action potential rate, abolishing weak Fröhlich condensation (Cavaglià and Tuszynski, 2025a). Protein-free lipid monolayers independently support two-dimensional solitary waves with action-potential-like properties (threshold excitation, all-or-nothing behavior, annihilation upon collision) (Shrivastava and Schneider, 2014), with adiabatic phase transitions during propagation (Shrivastava et al., 2015) describable by the Zel'dovich–von Neumann–Döring (ZND) detonation framework (Shrivastava, 2018). These wave-propagating properties are intrinsic to the lipid interface itself.

(iii) **A strong, composition-dependent dipole potential**. Oriented headgroup dipoles, ester linkages, and interfacial water produce an electrostatic dipole potential of hundreds of mV and local fields on the order of 10^9^ V/m (Cavaglià and Tuszynski, 2025a; Brockman, 1994), making the interface a phase-sensitive electrodynamic surface at which information is represented by spatiotemporal phase patterns. Ephaptic coupling modulates firing thresholds in a phase-dependent manner (Anastassiou et al., 2011; Buzsáki et al., 2012), so the membrane responds to the temporal structure, not merely the amplitude, of incident fields.

(iv) **Mesoscale compartmentalization**. Lipid rafts, cholesterol- and sphingolipid-enriched nanoscale domains, tune local phase sensitivity and coupling efficiency (Lingwood and Simons, 2010; Levental et al., 2020), functioning as resonant subunits of the interface.

(v) **Vicinal water as a coherence medium**. Water adjacent to membranes [vicinal or exclusion-zone (EZ) water] displays altered hydrogen-bond networks, increased effective viscosity, and modified dielectric response (Palacios et al., 2020; Pollack, 2013; Disalvo et al., 2015; Elton et al., 2020). Within UHF (Cavaglià and Tuszynski, 2025a, Postulate 2) it is an effective coherence medium that modulates EM field propagation, stores phase-sensitive energy (Arani et al., 1995), and is bidirectionally coupled to membrane state (Bagchi, 2011; Tyler et al., 2015), making the boundary a self-modifying surface whose properties depend on its own history: the dynamical memory the Markov blanket presupposes but does not explain.

(vi) **Multiscale boundary conditions**. Cerebrospinal fluid (CSF) occupies cavity-like spaces sustaining standing and traveling modes in the 0.1–30 Hz range, coupled to cortical slow oscillations (Fultz et al., 2019; Dreha-Kulaczewski et al., 2015; Nedergaard and Goldman, 2020); within UHF (Cavaglià and Tuszynski, 2025a, Postulate 4) its geometry links the mesoscale interface to whole-brain coordination. Endogenous electromagnetic fields drive write/read cycles at the interface (Cavaglià and Tuszynski, 2025a, Postulate 3), while ultra-weak photon emission may report interface state non-invasively (Cifra and Pospíšil, 2014; Nishiyama et al., 2024). The electrostatic energy dissipated during a cortical action potential suffices to drive weak Fröhlich-like condensation in the phospholipid dipole ensemble (Cavaglià and Tuszynski, 2025a; Fröhlich, 1968, 1970), so the interface is an active source of coherent radiation; the non-linear frequency-scaling cascade of UHF, from molecular GHz vibrations through kHz membrane solitons to Hz-band cortical rhythms, projects microscopic interface dynamics into the brain's macroscopic electromagnetic architecture (Cavaglià and Tuszynski, 2025a) (Table 1).

**Figure 1:**
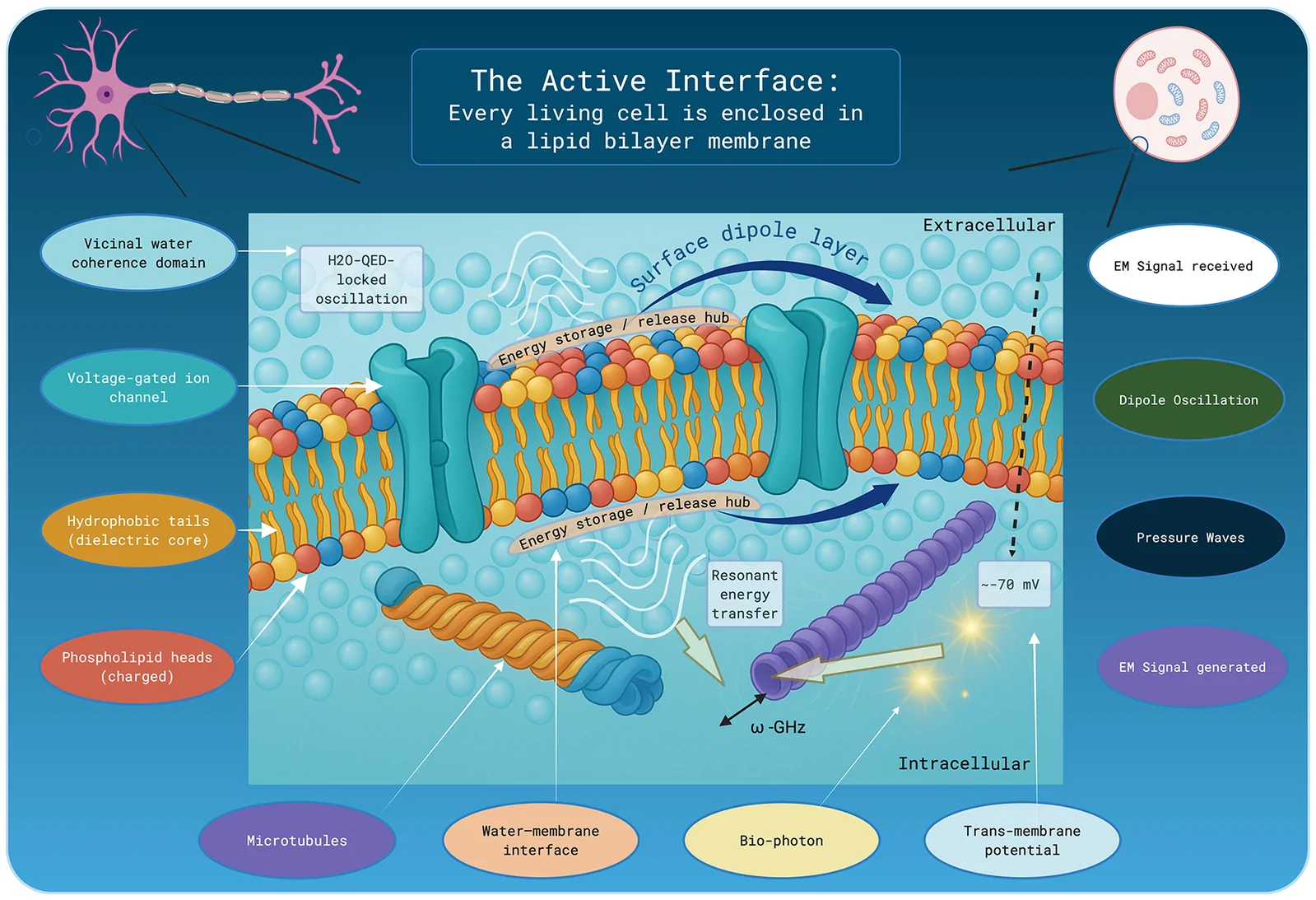
The active interface: membrane–water system as a resonant, phase-sensitive boundary. Left: cross-section of the lipid bilayer (charged headgroups, dielectric tails, voltage-gated channels, and intracellular microtubules) coupled to vicinal water coherence domains; the surface dipole layer stores and releases energy, supporting GHz biophoton emission under the transmembrane potential (~−70 mV). Right: the membrane in exclusion-zone water, with pressure waves, dipole oscillations, and EM signaling. This universal lipid membrane is the physical boundary that active inference formalizes as the Markov blanket.

**Table 1:** Systematic correspondence between active inference and active interference.

Dimension	Active inference	Active interference
**Boundary**	Statistical (Markov blanket)	Physical (resonant membrane–water interface)
**Information carrier**	Probabilistic states, belief distributions	Wave dynamics, phase patterns (Equation 1a)
**Core process**	Inference (Bayesian updating)	Interference (constructive/destructive superposition, Equation 2)
**Prediction**	Generative model encoding expected causes	Standing interference pattern biasing interface response
**Prediction error**	Mismatch between prediction and sensory input	Perturbation of attractor landscape by incoming wave
**Belief updating**	Revision of posterior distribution	Phase reconfiguration into new attractor state
**System–environment coupling**	Conditional independence	Resonant coupling
**Memory**	Updated prior distributions	History-dependent interface state (hydration, raft configuration, Equation 2c)
**Energy**	Free-energy minimization (info-theoretic)	Thermodynamic energy exchange at phase transition
**Falsifiable signature**	Prediction error signals in neural recordings	Phase-specific degradation under interface perturbation

This multilayered system is a physically instantiated, resonant interface that generates the dynamics statistical formalisms only describe. Its physical properties are not incidental to autonomy; they are constitutive of it.

## 3 From active inference to active interference

Sections 1 and 2 established that the Markov blanket does not specify the physical mechanisms of biological boundaries, and that the membrane–water interface possesses precisely the properties required to function as an active interface. We now articulate the central move: the transition from active inference to active interference.

We define *active interference* as the formation, stabilization, and reconfiguration of coherent interference patterns at resonant biological interfaces, through the superposition of internal and external oscillatory dynamics (Figure 2). The term captures three features. *Interference*: wave-like excitations superpose into constructive and destructive patterns that encode relational information in their phase structure, a substrate-neutral principle in which relative phase, not amplitude, carries the encoded relations. *Active*: the interface is not a passive reflector but an energetically sustained, non-linear medium that amplifies, gates, and reconfigures these patterns, driven by the energy dissipated during action potentials and sustained by the non-equilibrium thermodynamics of the membrane phase transition (Cavaglià and Tuszynski, 2025a; Heimburg and Jackson, 2005; Fröhlich, 1968). *Pattern*: the resulting structures are attractor-like configurations constrained by interface geometry, composition, and history, constituting the physical basis of what active inference describes as beliefs, predictions, and model updating.

**Figure 2:**
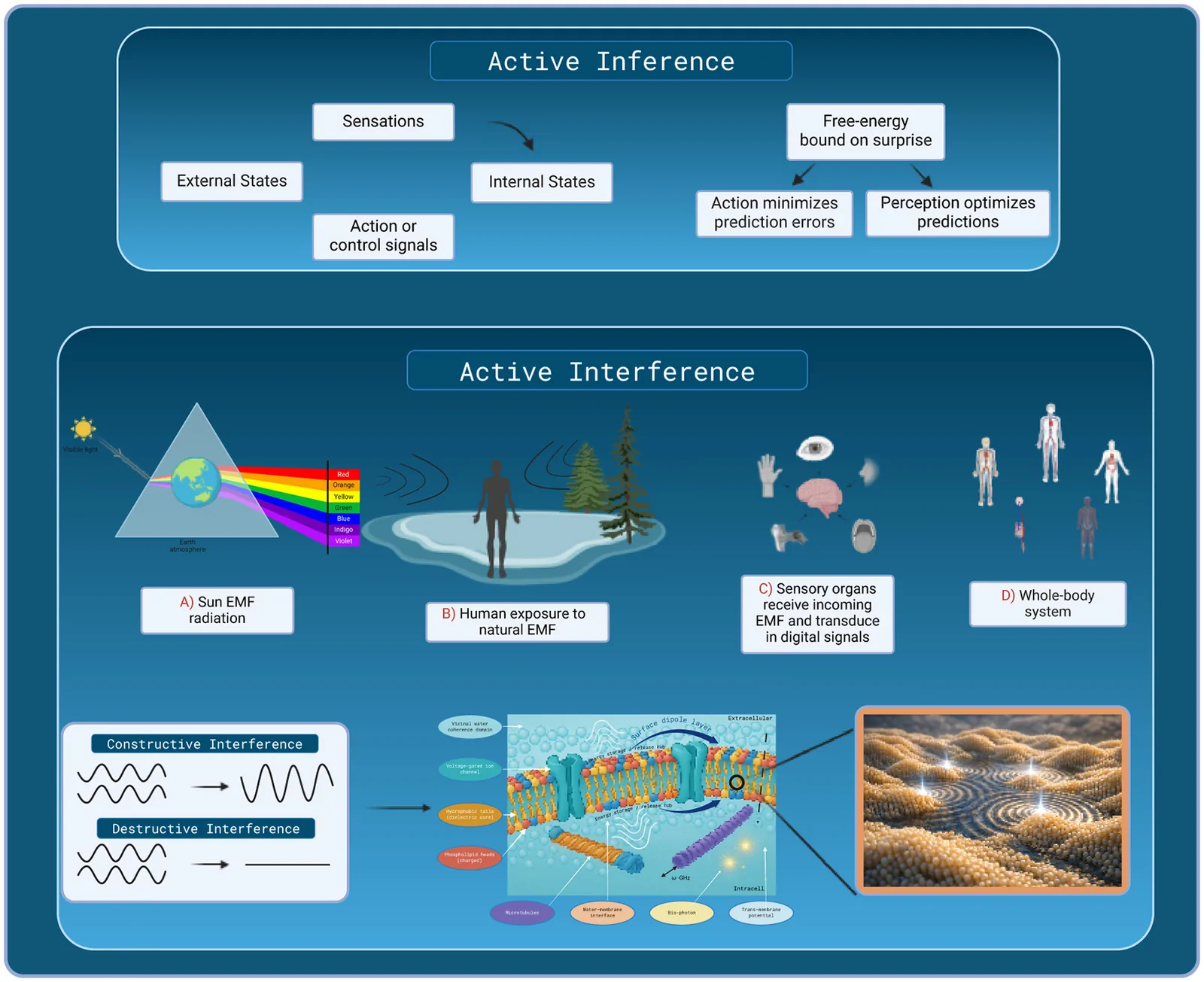
From statistical to physical boundaries. [**(Top)** panel]: the active inference architecture, in which external and internal states are coupled through sensory and active states across a statistical Markov blanket; perception optimizes predictions and action minimizes prediction errors, bounded by variational free energy. [**(Bottom)** panel]: the active interference framework, in which the same functional architecture is grounded in biophysics. Electromagnetic fields from the environment **(A)** impinge on the organism **(B)**, are transduced by sensory organs and interoceptive systems **(C,D)**, and processed at membrane–water interfaces through constructive and destructive wave superposition. The interference patterns formed at the lipid bilayer (center) constitute the physical substrate of what the statistical formalism describes as beliefs, predictions, and model updating.

### 3.1 A formal framework for interference dynamics

If the lipid interface can superpose, it can interfere; and if it can interfere, we are entitled to ask whether anything of the interference is left once the disturbances have passed. That question, made precise, is what it means for a membrane to remember.

Holography is the part of physics that answers it, and we will use its machinery almost unchanged (Gabor, 1948), extending the operational metrics of UHF (Box 3 of Cavaglià and Tuszynski, 2025a) and the account of membrane excitations as holographic image formation given in Cavaglià and Tuszynski (2025b). One thing has to be fixed first. In optics the recording medium and the light are different objects: the plate sits still, the beam goes through it. At a membrane there is no such division. The medium and the wave are the same material. The separation that optics is handed for free, we will have to earn.

**What is moving**. Let *S* ⊂ ℝ^2^ be a patch of the membrane–water interface, with **x** = (*x*_1_, *x*_2_) in the tangent plane; every field below lives on this surface, not along a line.

It is worth being precise about the main object of motion. Let ψ(**x**, *t*) be the displacement of the interface from equilibrium: mechanically the fractional change in area per lipid, electrically the headgroup polarization that must accompany it—a single variable, because electrostriction locks the two together. This is the quantity in which Heimburg and Jackson (2005) describe solitary propagation at a lipid interface. It is real, dimensionless and classical. It is not normalized, and |ψ|^2^ is proportional to an energy density; the symbol is borrowed for its wave and nothing quantum is meant by it.

Now watch one patch. It is doing three things at once. There is a fast oscillation at some frequency ω_0_. There is a slower swelling and fading of that oscillation's strength. And there is a drift that does not come back—the patch settles into a configuration slightly different from the one it started in. These are not three fields. They are one field on three clocks:


$$\begin{array}{l} \psi \left(x,t\right)=\psi_{eq}\left(x\right)+2Re\left[F\left(x,t\right)e^{i\omega_{0}t}\right]+\psi_{s}\left(x,t\right) \end{array}$$ (1)

with ψ_*eq*_ the equilibrium configuration, *F* the slowly varying envelope of the oscillation, and ψ_*s*_ the rectified part that survives once the carrier is averaged away. The clocks are ordered:


$$\begin{array}{l} \omega_{0}^{-1}\ll |F/\partial_{t}F|\ll \tau_{\psi } \end{array}$$

where τ_ψ_ is the relaxation time of the slow interfacial variables. This ordering is the whole trick. On the fastest clock ψ_*s*_ is frozen: it is a fixed medium and the oscillation runs through it. On the slowest, the oscillation is a blur with an energy: the medium never sees the wave, only what the wave leaves behind. Optics gets this by putting the plate and the beam in different places. We get it by putting them on different clocks. Where the ordering fails, everything downstream fails with it.

**Why the field may be complex**. We did not choose to write the oscillation as a complex number; we are entitled to. ψ is real, and *F* is its analytic signal, so:


$$\begin{array}{l} F\left(x,t\right)=A\left(x,t\right)e^{i\phi \left(x,t\right)} \end{array}$$ (1a)

is a representation rather than an assumption, and *A* and φ are a modulus and an argument rather than a split imposed by hand. A single scalar envelope is enough only if one mode dominates, and that is what Fröhlich condensation buys: when metabolic input outruns dissipation, the lowest collective mode takes most of the oscillatory energy (Fröhlich, 1968), and ω_0_ is its frequency. Under the ~20 MV/m gradient of the action potential, neighboring dipoles couple through their phase difference rather than their amplitudes, so it is φ, not *A*, that carries relational structure.

**What the interface does to a wave**. A hologram is not a record. It is an object that does something to light. The same must be true here, and it is the drift ψ_*s*_ that does it: a lasting change in local density changes the lateral compressibility κ, and it is that change—not the displacement itself—that a later wave encounters. Call the fractional change χ, and let *K* be the response kernel it induces:


$$\begin{array}{l} \chi \left(x,t\right)=\frac{\partial \ln\kappa }{\partial \psi }\psi_{s}\left(x,t\right),\\ F_{out}\left(x,t\right)=\int_{S}K\left(x,x^{\prime };\chi \right)F_{in}\left(x^{\prime },t\right)d^{2}x^{\prime } \end{array}$$ (1b)

The coefficient ∂lnκ/∂ψ deserves a moment, because it is the sole reason a stored displacement can ever be read. Here ∂lnκ/∂ψ cares about the change in the order of magnitude of compressibility as a function of change in membrane wave. It is largest where compressibility responds most steeply to density, which is to say at the chain-melting transition. The membrane must sit near its transition in order to record anything at all. Section 2(i) reports this empirically; here it follows from the formalism.

If the bilayer is thin compared with the transverse wavelength, *K* is diagonal and the whole operation collapses to a multiplication:


$$\begin{array}{l} K=\left[1+\chi \left(x,t\right)\right]\delta \left(x-x^{\prime }\right),\\ F_{out}\left(x,t\right)=\left[1+\chi \left(x,t\right)\right]F_{in}\left(x,t\right) \end{array}$$ (1c)

Read the grammar off this. The slow state is the operator; the fast envelope is what it operates on. *F* does not act on ψ − *F* is part of ψ, and the trace *H* below is a structure inside χ (A thick or multilamellar interface keeps *K* off-diagonal and acquires Bragg-like angular selectivity; we do not need that here).

**Holding a phase**. Two waves can be equally strong and still store nothing, if the phase between them wanders. The quantity that measures this is the complex degree of coherence; with $I_{1}\equiv |F_{1}{|}^{2}$ and $I_{2}\equiv \;|F_{2}{|}^{2}$,


$$\gamma_{12}(x)=\frac{{\langle F_{1}^{*}F_{2}\rangle }_{T}}{\sqrt{{\langle I_{1}\rangle }_{T}{\langle I_{2}\rangle }_{T}}},\;0\leq |\gamma_{12}|\leq 1$$ (1d)

where 〈·〉_*T*_ is a running average over a window *T* at the point **x**. Here is where the question of what is averaged gets settled. The numerator is averaged in the complex plane; the denominator over intensities. What survives is phase agreement with amplitude divided out: |γ_12_| = 1 is a relation held fixed across *T*, |γ_12_| = 0 is no relation at all. And the average runs over the history of the field at that point, which is all a membrane has access to. No ensemble is invoked, and neither stationarity nor ergodicity is assumed—which is fortunate, because a system that is learning is not stationary.

**Writing**. Let two coherent excitations arrive together:


$$\begin{array}{l} I\left(\text{x},t\right)=|F_{1}+F_{2}{|}^{2}=|F_{1}{|}^{2}+|F_{2}{|}^{2}+2A_{1}A_{2}\cos\left(\Delta \phi \right) \end{array}$$ (2)

The cross-term carries the phase relation, and the cross-term is what the interface keeps. Separating what it keeps from the flat background it also acquires,


$$\begin{array}{l} \chi \left(x\right)=\eta \left[{\langle I_{1}\rangle }_{T}+{\langle I_{2}\rangle }_{T}\right]+2Re\left[H\left(x\right)\right],\\ H(x)=\eta {\langle F_{1}^{*}F_{2}\rangle }_{T} \end{array}$$ (2a)

with η = λ ∂ ln κ/∂ψ collecting two conversions in series: λ, with which cycle-averaged energy drives the drift, and ∂ ln κ/∂ψ, with which the drift changes what a wave feels. *H* is the trace.

Three things follow, and none of them was put in by hand. First, 2*Re*[*H*] is exactly the cross-term of Equation 2, so *H* is the interference and nothing else. Second, *H* is complex while χ is real, and a real modulation cannot hold *H* without also holding H^*^: the conjugate that reading will require is forced on us, not inserted. Third, comparison with Equation 1d gives:


$$\begin{array}{l} H\left(x\right)=\eta \sqrt{{\langle I_{1}\rangle }_{T}{\langle I_{2}\rangle }_{T}}\gamma_{12}\left(x\right) \end{array}$$ (2b)

The trace is the coherence function itself, scaled by intensity and by the sensitivity of the medium. Memory at the interface is coherence that has been made material.

**How it accumulates**. The drift does not appear at once. It integrates the energy delivered to it and forgets at its own rate:


$$\tau_{\psi }\frac{\partial \psi_{s}}{\partial t}=-\psi_{s}+\lambda I(x,t),\;\psi_{s}(x,0)=\psi_{s0}(x)$$ (2c)

Notice that the drive is *I* and not *F*. This is not a modeling choice. It follows from Equation 1: the carrier averages to zero over a cycle, so only rectified energy ever reaches the slow variables. Writing is quadratic and reading is linear for this reason and no other.

Two consequences. The initial condition ψ_*s*0_ is whatever the interface was carrying beforehand, so each exposure updates a standing state rather than replacing it; and the average in Equation 2a is physically realized by Equation 2c as an exponentially weighted running average with kernel $e^{-s/\tau_{\psi }}/\tau_{\psi }$ and effective window *T* ≈ τ_ψ_. Because the integrator leaks, repetition and not single exposure writes a trace: contributions whose phase drifts between exposures lower |γ_12_| and are recorded weakly, while consistent ones accumulate. And τ_ψ_ is not free. It is the relaxation time of hydration shell, raft arrangement, composition and lateral tension (Section 2, iv–v), so the lifetime of a memory is something one measures.

How deeply the pattern is cut is the fringe visibility, and it takes its textbook form:


$$\begin{array}{l} V\left(x\right)=\frac{I_{\max}-I_{\min}}{I_{\max}+I_{\min}}=\frac{2A_{1}A_{2}}{A_{1}^{2}+A_{2}^{2}}|\gamma_{12}\left(x\right)| \end{array}$$ (2d)

*V* = 1 demands both equal amplitudes and full coherence; either failing sends it to zero. This is the exact content of the claim that the trace is built by averaging in the complex plane rather than over |*F*|^2^. It is |γ_12_|, not 〈*I*〉, that decides whether anything is written at all.

**Reading**. A cue arrives, $S_{in}\left(\text{x},t\right)=A_{S}e^{i\varphi_{S}}$, and by Equation 1c the interface simply multiplies it:


$$\begin{array}{c} F_{out}=[1+\chi ]S_{in}=[1+\eta (\underset{transmitted\;cue}{\underset{︸}{{\langle I_{1}\rangle }_{T}+{\langle I_{2}\rangle }_{T}}})]S_{in}\\ +\underset{reconstruction}{\underset{︸}{HS_{in}}}+\underset{twin}{\underset{︸}{H^{*}S_{in}}} \end{array}$$ (3)

Three terms, and they are the three outputs of any hologram. The first is the cue, attenuated. The second is the one that matters: cue with the reference, *S*_*in*_ = *F*_1_, and if *F*_1_ is quasi-stationary over *T* then *HS*_*in*_ = η〈*I*_1_〉_*T*_*F*_2_. Present one wave and the other comes back. Nothing in the membrane knows what a pattern is; recall is a consequence of multiplication and nothing more. The third term, $H^{*}S_{in}$, carries phase 2φ_1_ − φ_2_ and is the phase-reversed companion of the reconstruction. It is unavoidable whenever the recorded modulation is real, and it predicts that recall at a lipid interface never arrives alone.

A downstream reader that correlates the interface output with the cue returns the phase-sensitive inner product—now derived rather than posited:


$$\begin{array}{l} R\left(t\right)=\int_{S}H^{*}\left(x\right)S_{in}\left(x,t\right)d^{2}x \end{array}$$ (3a)

Peaks of |*R*(*t*)| are resonant recall. Because ψ_*s*_ carries its own time dependence, |*R*(*t*)| grows with consolidation and decays over τ_ψ_ once imprinting stops: recall strength depends on rehearsal history, not on the cue alone. The domain is fixed by physics rather than convention—*S* extends as far as the transverse coherence length and no further, since past that length the integrand averages to zero and a larger patch adds noise but no signal. Equation 3a is maximal when φ_*S*_ ≈ φ_*H*_ across the patch, and is formally the circular convolution of holographic reduced representations (Plate, 1995; Kanerva, 2009).

**Order across the patch**. Coherence in time is not yet coherence in space. The second is measured by the Kuramoto order parameter,


$$\begin{array}{l} r\left(t\right)=|\frac{1}{N}\overset{N}{\underset{k=1}{\sum }}e^{i\varphi_{k}\left(t\right)}| \end{array}$$ (4)

over the phases of *F* at *N* tiles of *S*, or at the macroscale over cortical nodes. Equations 2d, 4 are two independent conditions on a memory: |γ_12_| is phase agreement at one place across the exposure, *r*(*t*) is phase agreement across the surface at one instant. A trace is written only where the first holds, and read coherently only where the second does. Cognition, on this account, lives in metastable modulations of *r*(*t*) and its dwell times rather than in rigid phase-locking (Deco et al., 2017)—and interface perturbations should move it in ways no synaptic or connectomic account predicts.

**What we have assumed**. Five idealizations carry this, each standard in its own field and each capable of failing here. The three clocks of Equation 1 are well separated; one collective mode dominates; the interface is thin against the transverse wavelength; recording is linear, so λ is constant; and the domain is bounded by the coherence length. Each failure leaves a mark. Lose the separation of clocks and χ varies within a single exposure, blurring the trace as it forms. Add competing modes and *V* degrades while *r*(*t*) does not. Thicken the interface and recall acquires angular selectivity. Saturate λ and the reconstruction distorts harmonically. Overrun the coherence length and Equation 3a gains noise but no peak. Because every perturbation of the interface acts on ψ, and ψ sets η, τ_ψ_ and the coherence length together, the manipulations of Section 6.2 reach every quantity in Equations 1–4 along a route we can write down.

**Computational signature**. Because active interference is a physical specification of active inference, its dynamics should reproduce the statistical observations of inference, and should do so under the storage the formalism actually describes. We therefore cast four architectures within a single inference model and ask what each predicts on identical tasks. Each is given a bank of patterns and, from a partial and noisy cue, performs one step of Bayesian inference: a uniform prior, a likelihood, and a posterior from which the most probable pattern is read and scored. Prior, posterior update and scoring are identical throughout, so any difference comes from the mechanism alone. Storage is physical. Every trace is superposed into the single real modulation χ of Equation 2a and read by the single multiplication of Equation 1c, so that holding more traces in one medium costs crosstalk rather than nothing. Interference in the holographic condition writes each trace against its own reference carrier and demodulates there, which is how the three terms of Equation 3 are separated; the on-axis condition omits the carrier, so that the transmitted cue and the twin overlap the reconstruction and cannot be removed. Hopfield holds a single weight matrix. Active inference applies a Gaussian likelihood to templates retrieved from the same χ, and so pays the same crosstalk. Two further lines are references rather than candidates: the matched-filter bound, which applies that likelihood to clean templates and is the optimum available under per-slot access, and the list model of the earlier version of this analysis, in which each trace occupies a separately addressable slot and bank size costs nothing (Figure 3).

**Figure 3:**
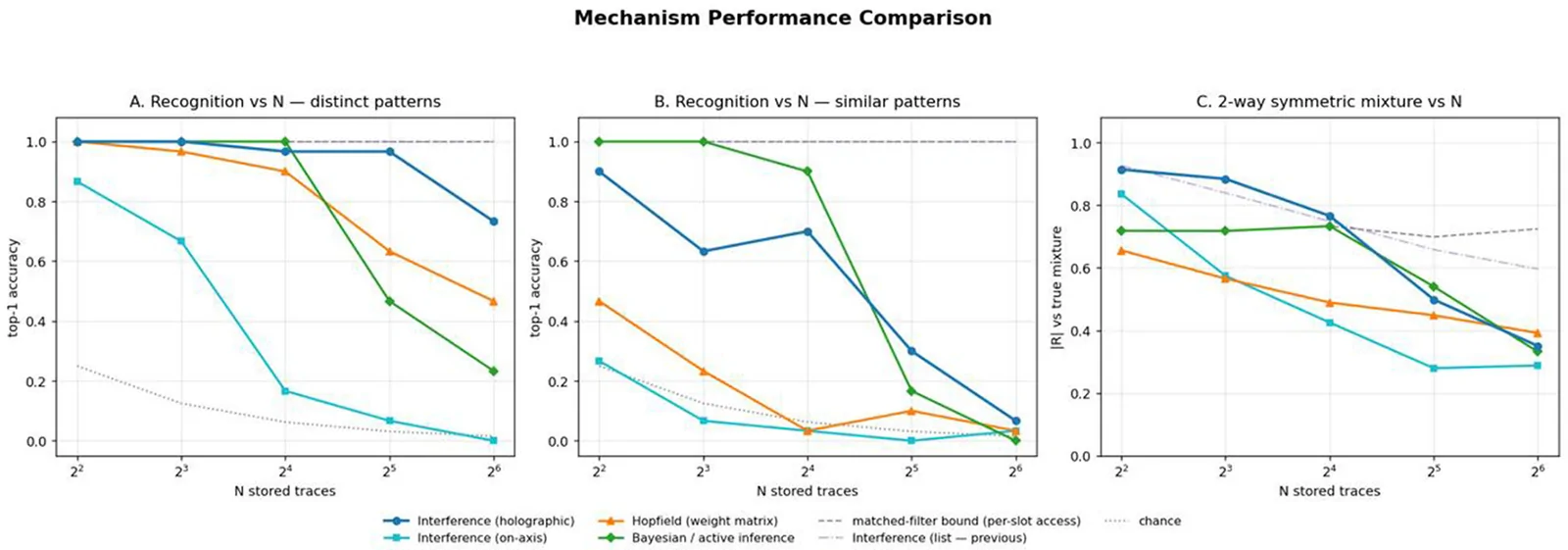
Mechanism performance comparison under physical storage. Four architectures in one identical free-energy framework, with the same prior, posterior update and scoring, so that differences come from the mechanism alone. Storage is physical: traces are superposed into a single real modulation χ (Equation 2a) and read out by one multiplication (Equation 1c), so bank size incurs crosstalk. Interference (holographic) writes each trace against its own reference carrier and demodulates there; on-axis omits the carrier, so the transmitted cue and conjugate term of Equation 3 overlap the reconstruction; Hopfield holds one weight matrix, ξ = sign(Re F); Bayesian/active inference applies a Gaussian likelihood to templates retrieved from the same χ. The matched-filter bound (gray dashed) applies that likelihood to clean templates, the optimum under per-slot access, and since the holographic readout computes the same inner product the gap between them is the cost of sharing one medium. Previous list model (purple dash-dot): separately addressable slots, no capacity cost. Gray dotted, chance. **(A)** Well-separated patterns. Inference tracks the bound at low load and departs as the medium fills, the zero-crosstalk limit of Section 3.2, while interference degrades more gracefully (0.97 against 0.47 at *N* = 32). **(B)** Patterns sharing coarse structure and differing only in fine phase: both phase-sensitive mechanisms hold where Hopfield's sign-overlap collapses. **(C)** Two-way mixtures, where a nearest-template rule has no state between stored patterns; here the gray line marks per-slot access rather than an upper bound, since reconstruction quality depends on how sharply the posterior concentrates and not on identification alone. 64 × 64 band-limited complex fields; cues half-masked, complex Gaussian noise σ = 0.8; 30 seeds. Hopfield on a 32 × 32 downsample.

Under physical storage the ordering changes with load rather than with task, and that is the result. In panel A every architecture sharing one medium degrades as the bank fills, while the matched-filter bound and the list model, both granted separately addressable slots, do not: the flat lines are the signature of per-slot access and not of a better mechanism. Among the physical architectures the holographic readout degrades most slowly, holding 0.97 at *N* = 32 where active inference on the same medium has fallen to 0.47 and Hopfield to 0.63, and 0.73 at *N* = 64 against 0.23 and 0.47. Active inference tracks the bound exactly while the medium is nearly empty and departs from it as crosstalk accumulates, which gives the claim of Section 3.2 a quantitative form: inference is what interference looks like in the zero-crosstalk limit, and that limit is one a physical medium leaves as soon as it holds more than a few traces. Panel B discriminates between likelihood rules rather than between capacities. Its patterns share coarse structure and differ only in fine phase, and the sign-overlap rule discards exactly what separates them: Hopfield is at 0.47 with four traces stored and does not recover. Both phase-sensitive mechanisms survive there, and neither dominates the other—active inference is the more accurate while the medium is nearly empty and the holographic readout the more robust once it fills. Panel C measures reconstruction of a two-way mixture, where a nearest-template rule has no state genuinely between stored patterns; the holographic condition leads at low load, at 0.91 with four traces stored, and the architectures converge as the medium fills. The on-axis condition is the control that isolates the reference carrier. Recording without angular separation leaves the transmitted cue and the twin term of Equation 3 superposed on the reconstruction with no geometry available to reject them, and recognition falls from 0.87 with four traces stored to 0.17 at 16 and to 0 at 64. The twin term is therefore not a formal inconvenience but a measurable cost, and the carrier is what turns a medium that merely holds a trace into one that can be addressed. What the demonstration does and does not do should be stated plainly. It implements the storage and the readout of the formalism—one shared modulation, one multiplication, orders separated by carrier—and not the interfacial physics that produces them: there is no membrane in it, no compressibility and no consolidation dynamics. What it establishes is accordingly narrower than the framework and load-bearing for it. A single medium holding superposed traces, read by one multiplication, sustains content-addressable recall well past the point at which the same medium read by other rules has failed, and its capacity limit follows from the geometry of the patch rather than from any choice made in the model. The limits of substrate-independent emulation are taken up in Section 7.

### 3.2 Inference as a limiting case of interference

Active interference is not a competitor to active inference but a specification of its physical implementation: the relationship is one of containment, not opposition. Table 1 sets out the systematic correspondences. The decisive implication is that inference does not generate biological agency, it traces it: the organism does not compute a posterior, it resonates into a new configuration. Active inference characterizes adaptive behavior through information-theoretic quantities (free energy, surprise, and posterior beliefs); active interference specifies the physical process from which those quantities are abstracted. The information-theoretic description is the more restricted of the two, being what remains once the phase structure of the interface is coarse-grained into conditional-independence relations and its thermodynamics re-expressed as a divergence between probability densities. Figure 3 gives the containment a quantitative form. An architecture performing inference on the same shared medium tracks the optimum available under separately addressable storage while that medium is nearly empty, and departs from it as crosstalk accumulates: inference is what interference looks like in the zero-crosstalk limit.

Read this way, active inference is a special case in a precise sense, arising under two reductions: spatially, when the physical membrane–water boundary is replaced by a statistical Markov blanket (Biehl et al., 2021; Raja et al., 2021); and thermodynamically, when the non-equilibrium dynamics that sustain coherence are redescribed as minimization of a Kullback–Leibler divergence rather than as maintenance of evolutionary entropy at a dissipative interface (Section 4). The relationship parallels wave and ray optics: geometric optics is the limit of wave optics in which interference structure averages out, leaving discrete trajectories. Likewise, the belief-level account is what active interference looks like once relative phase, the carrier of relational information, has been integrated away. Free-energy minimization is then not a computation the organism performs but the appearance, in information-theoretic coordinates, of a system sustaining coherent interference at its resonant interfaces, and the broader framework recovers the same adaptive phenomena (anticipation, error correction, and pattern completion) without positing a representational layer or internal generative model. The organism need not minimize free energy in any computational sense; it need only sustain coherent interference, of which free-energy minimization is the statistical shadow.

## 4 Interference, holography, and form-in-action

The transition from inference to interference entails a shift in the concept of information. That information is carried by distinguishable physicochemical states rather than by symbols manipulated over them is a thesis Natural Computing has developed for several decades (Rozenberg et al., 2012); what follows specifies it for the case of a living membrane. In representational frameworks, information is a quantity, symbols or probability distributions standing for features of the world. In active interference it is reconceived as form-in-action: a dynamical structure emerging from the interaction between energetic flows and resonant boundaries, existing only insofar as it is sustained by ongoing physical processes. This aligns with the EMI model (Firaux et al., 2025), which treats information as intrinsic to the system's dynamical organization, inseparable from the energy that sustains it. Biological “representations” are thus not internal copies stored in neural circuits but transient interference patterns distributed across the membrane–water interface and whole-brain function, whose stability and accessibility depend on phase relationships among oscillatory processes. The information encoded is relational, distributed, and content-addressable (Equations 2, 3), matching the definition of holographic computation in UHF (Box 1 of Cavaglià and Tuszynski, 2025a): interference-based coding, distributed redundancy, phase-matching recognition, and write/read cycles via EM–hydration coupling.

This reconception has thermodynamic consequences. The free energy principle motivates surprise minimization by appeal to a thermodynamic intuition: that entropy increases, or at best remains constant, in isolated systems, so that living systems must resist this tendency (Friston, 2010). The intuition does not transfer. Living systems are neither isolated nor closed but open and dissipative, held far from equilibrium by continuous entropy export (Nicolis and Prigogine, 1977; Davies et al., 2012), and for such systems no general principle requires the minimization of a thermodynamic potential. The variational free energy minimized under active inference is in any case a Kullback–Leibler divergence, not a thermodynamic one. If the boundary is a statistical Markov blanket rather than a physical membrane, the mechanism managing entropy remains unspecified. Active interference addresses this directly: the membrane–water interface is a dissipative boundary sustaining order through energy throughput, phase-coherent interference maintained by EM–hydration coupling (Equations 1–4), not by minimizing an informational divergence.

The appropriate entropy for such open, cyclic systems is not Boltzmann–Gibbs entropy but evolutionary entropy *H*_*ev*_ (Demetrius, 1997, 2013, 2024), which quantifies the robustness of metabolic cycling far from equilibrium. *H*_*ev*_ is unrelated to the consolidated interference trace *H*(x) of Equation 2a, despite the shared letter. In Demetrius's directionality theory the growth rate decomposes as *r*_*g*_ = Φ + *H*_*ev*_, where Φ is reproductive potential and *H*_*ev*_ the entropy of the system's transition statistics per unit cycle time, an information rate rather than a static count of microstates. It incorporates resources and population size, the features distinguishing living systems, recovering Boltzmann–Gibbs entropy when the dynamics become memoryless and degrees of freedom unbounded. Selection among competing steady states, here competing patterns of interface oscillation, follows an entropic selection principle (Demetrius, 1997, 2013, 2024):


$$\begin{array}{l} \left(-\Phi +\gamma /M\right)\Delta H_{ev}\;\geq \;0 \end{array}$$ (5)

where γ is the temporal correlation of resource supply, M the effective ensemble size, and Δ*H*_*ev*_ the difference in evolutionary entropy between variant and incumbent. The inequality in Equation 5 states a condition rather than a tendency, and the sign of the prefactor is what carries its content: variants of lower evolutionary entropy are favored under a bounded, weakly correlated supply, and variants of higher entropy under a sustained supply with large M, so that the same system selects in opposite directions under different resource regimes. The decomposition, the conditions under which it holds, and its limiting behavior are derived in full by Demetrius (1997), Demetrius (2013), and Demetrius (2024), whose results we adopt rather than rederive. In the large-size limit (M → ∞) this reduces to a self-organizing principle: under a sustained energy source, the system settles into states that maximize evolutionary entropy. Such joint dependence on resources and size has no analog in equilibrium thermodynamics, nor in a variational free energy over probability densities alone. What active inference describes as surprise minimization is, at this level, the maintenance of evolutionary entropy at a dissipative interface, the same process in information-theoretic rather than thermodynamic coordinates; its constructs are recovered once that resource- and size-dependent thermodynamics is coarse-grained into a divergence between probability densities.

These dynamics have direct consequences for perceptual organization, illustrated by bistable perception (Figure 4). Stimuli such as the Necker cube and the Rubin vase support two mutually incompatible organizations from a single, unchanging input. Here these are competing phase-coherent configurations across membrane–water interfaces, each a thermodynamically sustainable attractor; switching reflects fluctuation-driven transitions in the phase landscape, internal dynamical reorganization rather than changes in sensory evidence (Deco et al., 2017). This resolves the binding problem physically: features of the same object share a common phase relationship within the global interference pattern *H*(x), features of different objects occupy distinct configurations, and the Kuramoto order parameter *r*(*t*) (Equation 4) quantifies binding at any moment. Binding is achieved not by a central integrator but by the geometry of wave superposition at a resonant boundary, formally equivalent to holographic multiplexing, where multiple patterns coexist in one medium distinguished solely by phase (Cavaglià and Tuszynski, 2025a; Gabor, 1948; Plate, 1995); coherence-based integration studies support the primacy of phase over amplitude in perceptual unity (Fries, 2005; Plankar et al., 2013). Sensory gating should therefore be phase-dependent: the alpha cycle (8–12 Hz) may be a periodic opening and closing of the interface's sensitivity window, a write–read alternation (Equations 2, 3) in which high-excitability phases permit constructive interference while low-excitability phases suppress it. Empirical data confirm this: pre-stimulus alpha phase predicts conscious detection of near-threshold stimuli (Spaak et al., 2014; Samaha and Postle, 2015), and flickering stimuli selectively enhance or disrupt perception depending on phase alignment with endogenous rhythms (Lakatos et al., 2008). Both effects are phase-specific, not amplitude-specific, yielding a falsifiable prediction: the signatures of perceptual switching should be phase-specific (bicoherence, phase-gradient directionality, and metastable dwell-times) rather than amplitude-specific (spectral power), degrading selectively under interface perturbations that compromise phase coherence without eliminating neural activity.

**Figure 4:**
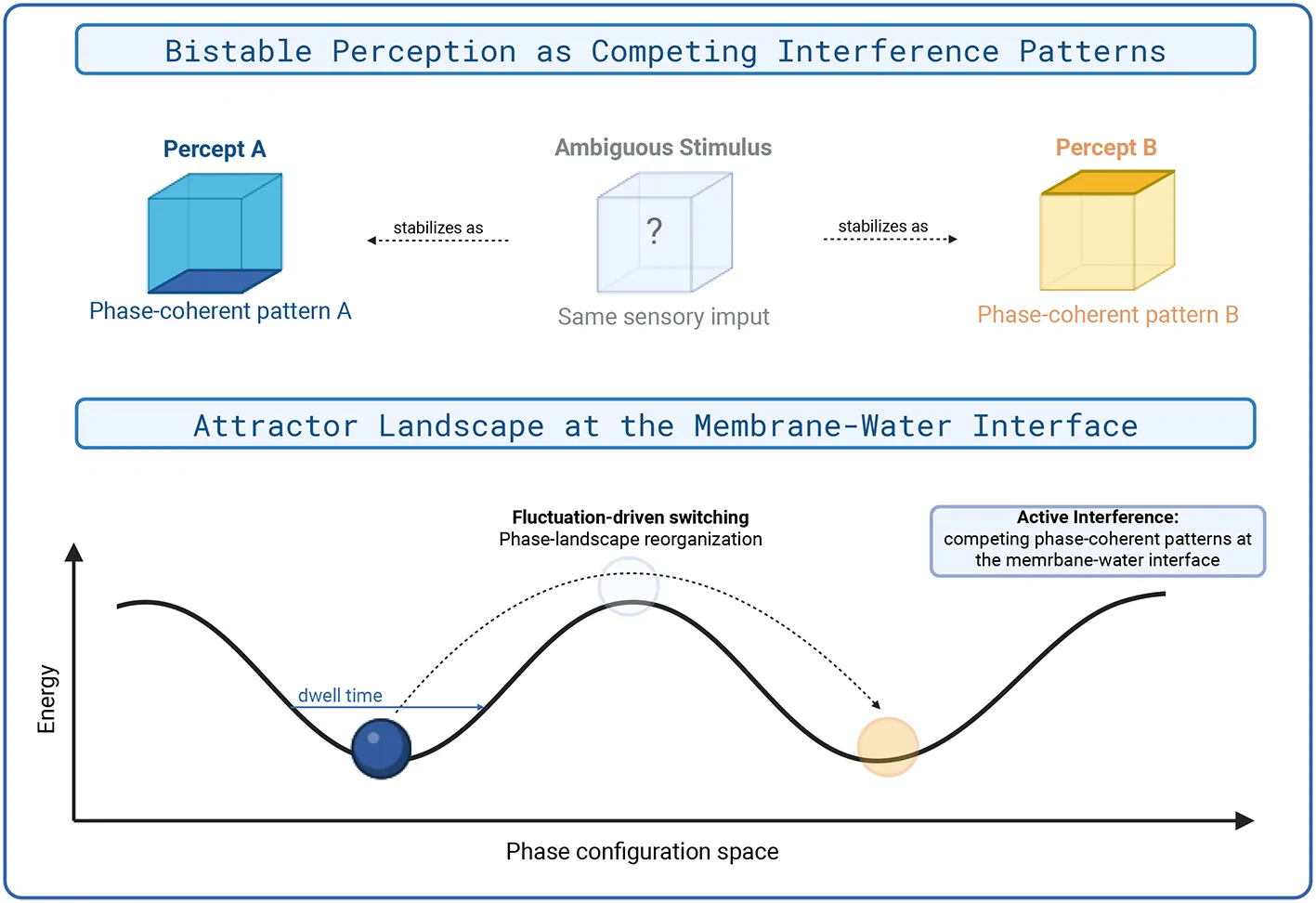
Bistable perception as competing interference patterns. **(Top)**: an ambiguous stimulus (e.g., Necker cube) supports two mutually incompatible percepts (A and B), each corresponding to a distinct phase-coherent pattern stabilized across the membrane–water interface. The same sensory input resolves into one or the other configuration depending on the current state of the phase landscape. **(Bottom)**: the corresponding attractor landscape in phase configuration space. Each percept occupies a local energy minimum; perceptual switching corresponds to fluctuation-driven transitions between wells, with dwell time reflecting attractor depth. Active interference is the dynamical process by which competing phase-coherent patterns at the membrane–water interface settle into one stable configuration—and periodically reorganize into the alternative.

### 4.1 Anticipatory dynamics through phase alignment

Active inference explains anticipation through minimization of expected prediction error via a hierarchical generative model (Friston, 2010). Active interference offers a more parsimonious account: anticipation emerges directly from the physics of phase-gated resonance, without an internal model. An interference pattern *H*(x) consolidated through repeated imprinting (Equation 2c) establishes a temporal filter; because superposition is phase-sensitive, the response *R*(*t*) to incoming signals (Equation 3a) depends on phase alignment, amplifying in-phase inputs and suppressing out-of-phase ones. The interface operates as a resonant clock whose history-dependent phase configuration determines when and which signals are transmitted. Neurophysiology confirms this: low-frequency oscillations in sensory cortex entrain to rhythmic stimulus streams, aligning high-excitability phases with expected event timing and producing anticipatory gain through phase alignment alone (Lakatos et al., 2008; Schroeder and Lakatos, 2009; Stefanics et al., 2010; Leszczynski and Schroeder, 2019).

This operates across scales, through a hierarchy of endogenous sensory clocks whose rhythms entrain cortical phases and establish cyclic sensitivity windows. The 3–4 Hz saccadic rhythm structures visual sampling by resetting occipital alpha and medial temporal theta at each fixation; the 0.1 Hz cardiac and 0.05 Hz gastric rhythms gate interoceptive and affective processing at slower timescales. In each case the system does not predict event timing by computing a probability distribution; it pre-configures its phase structure to be maximally receptive when signals are expected. Brain–body coupling studies support this hierarchy, with the slowest shared resonance frequency decreasing with spatial distance between coupled oscillators (Young et al., 2022; Richter et al., 2017; Hunt and Schooler, 2019), consistent with the EMI model (Firaux et al., 2025), which treats distributed brain–body oscillatory coupling as the substrate for coherent attractor formation extending functionally beyond the skull. Prediction error minimization thus corresponds to adjusting the interface's phase configuration to maximize constructive interference with recurrent inputs; a precise prediction is an interference pattern producing high-amplitude resonant matching; surprise is phase mismatch driving reconfiguration of the attractor landscape; and precision weighting is the degree of local phase coherence *r*(*t*) (Equation 4), which amplifies phase-matched inputs and suppresses mismatched ones rather than constituting a separate computation. What predictive processing describes computationally, active interference implements physically.

## 5 Convergent validation: from biophysics to engineering

The core principle of active interference, computation via wave interference at lipid interfaces, has been independently approached from five converging directions. None alone establishes the framework; together they constitute a cumulative case difficult to attribute to artifact.

The theoretical foundation is UHF (Cavaglià and Tuszynski, 2025a) and the EMI model (Firaux et al., 2025), which characterize the membrane–water–CSF system as a phase-sensitive interference processor and connect molecular interface dynamics to cognition through operational metrics: global phase order *r*(*t*), bicoherence, phase-gradient directionality, and Raman and fluorescence-lifetime imaging microscopy (FLIM) reporters. The energetic feasibility of Fröhlich-like condensation (Fröhlich, 1968, 1970) under action-potential driving has been quantitatively established (Cavaglià and Tuszynski, 2025a), grounding the write/read cycle of Equations 2, 3. That such condensation is thermally accessible without external optical driving has now been shown experimentally: Perez-Martin et al. (2025) found that thermal energy alone activates collective low-frequency vibrational modes in a light-harvesting protein, with Q-factor evolution characteristic of a phase transition. The basis for vicinal water as a coherence medium derives from Del Giudice et al. (1986), Del Giudice et al. (1988), and Arani et al. (1995), who showed that quantum electrodynamic (QED) interactions in polar liquids produce coherent domains stabilized by amphiphilic surfaces, the same framework that accounts for sub-thermal coupling of environmental fields to biological interfaces.

At the engineering level, Shrivastava (2024) demonstrated a lipid monolayer device implementing logical operations, artificial neurons, and linear classifiers through wave interference alone, without ion channels or digital representations; solitonic threshold behavior and pulse annihilation confirm the Heimburg–Jackson dynamics (Heimburg and Jackson, 2005; Shrivastava et al., 2015), while the bias-voltage mechanism implements history-dependent excitability, the physical counterpart of an active-inference prior.

Recent biomimetic work extends this to biologically relevant preparations. Podar et al. (2025) showed that electromechanically induced membrane restructuring enables learning and memory: repeated stimulation drives the bilayer into a non-equilibrium steady state where composition-dependent restructuring modulates channel conductance in a history-dependent manner, a physical implementation of synaptic plasticity requiring no protein synthesis. The same group showed droplet interface bilayers function as physical reservoir computers exceeding 99% classification accuracy (Mohamed et al., 2024), exploiting the nonlinear fading-memory dynamics of Equations 1–4. Oscillatory field exposure produces bistable hysteretic curvature states functioning as physical memory (Lavrentovich et al., 2025; Kageyama et al., 2025), and lipid rafts polarize directionally in applied fields, their disruption abolishing field-directed cellular responses (Young et al., 2022), a concrete route through which extremely low frequency (ELF) fields modulate membrane state at sub-thermal intensities.

A fifth line comes from colloidal physics. Chiolerio et al. (2026) demonstrated significant phase correlations between shielded, physically separated ferrofluid aliquots, a system whose geometry (amphiphilic coating, dipolar medium) is the minimal analog of the membrane–water interface; correlations persist for ~100 h, are distance-independent, and violate classical joint probability. The account invokes spontaneous symmetry breaking and coherent boson condensation in quantum field theory (QFT) (Blasone et al., 2011), the tradition of Del Giudice et al. (1986), Del Giudice et al. (1988), and Arani et al. (1995). A phase-coherent state analogous to the interference trace *H*(x) of Equation 2a can thus be imprinted in an amphiphilic interface, maintained for biologically relevant timescales, and read out at room temperature, supporting the write/read cycle independently of any biological preparation.

The five programs, biophysical (Cavaglià and Tuszynski, 2025a; Firaux et al., 2025), engineering (Shrivastava, 2024), neuromorphic (Podar et al., 2025; Mohamed et al., 2024), electromechanical (Lavrentovich et al., 2025; Kageyama et al., 2025), and colloidal (Chiolerio et al., 2026), converge on phase-coherent interference at amphiphilic interfaces as physically realizable, thermally robust, and computationally productive, given an amphiphilic boundary, a dipolar aqueous medium, and collective dynamics near a phase transition. The principal remaining gap is direct neural validation: the neuromorphic results (Podar et al., 2025) partially close it but lack biological synaptic architecture, and the UHF roadmap (Table 5 of Cavaglià and Tuszynski, 2025a) specifies the decisive steps (cholesterol depletion, anesthesia, D_2_O substitution), whose predictions are stated in Section 6.2.

## 6 Discussion

### 6.1 Inference, interference, and the relational nature of biological information

Under the FEP, the Markov blanket lets internal states parameterize a variational density over external causes, a structure readable as probabilistic modeling (Hohwy, 2013; Friston, 2019; Ramstead et al., 2023). A more precise characterization follows once we specify the physical substrate (Section 2) and the wave dynamics that play out across it (Section 3). The conditional independence captured by the blanket is a relational property of the coupled system as a whole, not an ontological wall between a self-contained interior and an independent exterior. What the interface encodes is therefore not an internal copy of the world but something constitutively dependent on the ongoing exchange between the domains it mediates. Renati and Madl (2025) make the same point from a quantum field theoretic perspective: in living systems structure and function are co-substantiated, and medium and message cannot be prised apart. On this view the membrane is not a place where representations are stored but a medium through which coherent electromagnetic interference patterns are continuously generated and reconfigured (Firaux et al., 2025). What the FEP calls updating a generative model is, biophysically, the reshaping of standing and traveling wave patterns across the bilayer and its dipolar field, so that inference becomes a real-time, spatially distributed process of wave-mediated coordination rather than computation over symbols. Gödel (1931) incompleteness theorems sharpen the analogy. A system embedded in a larger dynamical whole cannot build a complete internal model of the environment it belongs to. Its best adaptive strategy is therefore not to represent that environment exhaustively, but to remain in coherent electromagnetic coupling with it. The membrane does not seal the system off from the world; it is the dynamic locus at which inside and outside are co-specified through ongoing constructive and destructive interference. The same locus has been reached from cell biology: a recent revision of cell theory places the plasma membrane at the epicenter of cellular bioelectromagnetic fields and identifies it as the organizer of the senome, the sum of a cell's sensory perceptions held as a field and translated into biochemical form upstream of the epigenome and genome (Baluška and Miller, 2018; Miller et al., 2020; Baluška, 2025). The two accounts are complementary. That literature establishes the membrane as the organizing locus of cellular information, while the formalism of Section 3 offers a mechanism by which such a layer might write and read. They converge again on epistemic limits, since the claim that cell-based observers cannot obtain observer-independent knowledge of external reality (Miller et al., 2026) is the conclusion reached above from Gödel's theorems by another route.

So construed, biological autonomy is constituted by the physical properties of the system–environment interface, not merely described by a statistical partition laid over it. The picture resonates with Levin (2024) account of multiscale collective intelligence, in which agency belongs to any system that can sustain goal-directed information processing. What grounds the continuity of agency across scales is the universality of the membrane–water interface itself: the interface is everywhere the same, and only its complexity and hierarchical embedding change as one moves from single-cell bioelectric signaling through neural ensembles to whole-brain, CSF-mediated coordination (Cavaglià and Tuszynski, 2025a).

### 6.2 Falsifiable predictions

The framework generates predictions distinct from standard active inference, testable with existing methods. The critical prediction is that manipulations of phase structure at the membrane–water interface will degrade cognitive signatures more selectively than manipulations of amplitude. Specifically: (i) cholesterol depletion should reduce bicoherence and phase-gradient directionality by more than it reduces spectral power, against a vehicle-treated control, and is falsified if power falls in proportion or more; (ii) graded anesthesia should produce concerted, dose-graded decreases in phase order, bicoherence and Raman/FLIM markers (Heimburg and Jackson, 2007; Purdon et al., 2013), against a sedation-matched non-anesthetic, and is falsified if these dissociate across dose; (iii) D_2_O substitution should reduce phase-sensitive coupling between interface reporters and electrophysiological metrics while sparing firing rate and spectral power, against an osmolarity-matched H_2_O control; (iv) phase-scrambling with power preserved should abolish the predicted spectro-phase co-variation, a decisive negative control that fails if the co-variation persists. Each is executable with current human electrophysiology and imaging.

## 7 Conclusion

The transition from active inference to active interference is not a play on words. It marks a shift in explanatory ontology: from statistical partitions to physical interfaces, from probabilistic representations to dynamical phase patterns, from computational metaphors to wave superposition at resonant boundaries. This does not invalidate active inference; it grounds it in a biophysical mechanism and opens it to a new class of falsifiable predictions.

Three propositions summarize the framework. First, biological autonomy is constituted by physically instantiated, resonant interfaces. In the cases considered here these are lipid membranes coupled to vicinal water and to endogenous electromagnetic fields. Their nonlinear, excitable, history-dependent properties generate the dynamics that statistical formalisms only describe. Second, biological information is form-in-action, not a static representation of external causes. Third, this principle is already independently validated biophysically (UHF–EMI) and through engineering (Shrivastava, 2024), with a formal framework (Equations 1–4) connecting interface physics to the information-theoretic quantities of active inference.

Whether the framework withstands experimental scrutiny remains open; what it offers immediately is a sharply framed, testable account of how energy, mass, and information converge at the boundaries of living systems.

This reframing carries an ontological corollary. In an engineered system, hardware and software are separable: the same algorithm runs on any substrate, and a simulation of a physical process is not that process. At a resonant biological interface this separation collapses. The dynamics and what they encode are two faces of a single physical event, and the membrane therefore does not operate upon a representation: it is the configuration it instantiates. Chemical Artificial Intelligence makes the same wager on different material, treating molecules and macromolecules as hardware and the non-equilibrium processes of the liquid phase (chemical reactions, diffusion, migration, convection, chemical waves) as software (Gentili, 2025). Active interference contributes one further process to that list and gives it a boundary. The two frameworks also share a consequence that can be tested: substrate-independent emulation should reproduce what depends only on the computational rule and fail on what depends on the physical phase state itself. The first is illustrated by Figure 3, where rule and storage alike are emulated digitally; the second is addressed by the perturbations of Section 6.2. The distinction between simulating and instantiating a physical system, recently pressed in the philosophy of computation (Lerchner, 2026), thereby acquires a concrete biophysical locus: phase is not a code read off the substrate, but a state the substrate occupies.

## Data Availability

The datasets presented in this study can be found in online repositories. The names of the repository/repositories and accession number(s) can be found below: https://doi.org/10.5281/zenodo.20735753.

## References

[B1] Anastassiou C. A. Perin R. Markram H. Koch C. (2011). Ephaptic coupling of cortical neurons. Nat. Neurosci. 14, 217–224. doi: 10.1038/nn.2727 21240273

[B2] Arani R. Bono I. Del Giudice E. Preparata G. (1995). QED coherence and the thermodynamics of water. Int. J. Mod. Phys. B 9, 1813–1841. doi: 10.1142/S0217979295000744

[B3] Bagchi B. (2011). Water in Biological and Chemical Processes. Cambridge: Cambridge University Press.

[B4] Baluška F. (2025). Cognitive cells: from cellular senomic spheres to Earth's biosphere. Biol. Theory [Preprint]. doi: 10.1007/s13752-025-00489-5

[B5] Baluška F. Miller W. B. Jr. (2018). Senomic view of the cell: senome versus genome. Commun. Integr. Biol. 11, 1–9. doi: 10.1080/19420889.2018.1489184 30214674 PMC6132427

[B6] Barrett L. F. (2017). The theory of constructed emotion: an active inference account of interoception and categorization. Soc. Cogn. Affect. Neurosci. 12, 1–23. doi: 10.1093/scan/nsx060 27798257 PMC5390700

[B7] Barrett L. F. Simmons W. K. (2015). Interoceptive predictions in the brain. Nat. Rev. Neurosci. 16, 419–429. doi: 10.1038/nrn3950 26016744 PMC4731102

[B8] Bhat A. Parr T. Ramstead M. J. D. Friston K. J. (2021). Immunoceptive inference: why are psychiatric disorders and immune responses intertwined? Biol. Philos. 36:27. doi: 10.1007/s10539-021-09801-6 33948044 PMC8085803

[B9] Biehl M. Pollock F. A. Kanai R. (2021). A technical critique of some parts of the free energy principle. Entropy 23:293. doi: 10.3390/e23030293 33673663 PMC7997279

[B10] Blasone M. Jizba P. Vitiello G. (2011). Quantum Field Theory and its Macroscopic Manifestations: Boson Condensation, Ordered Patterns, and Topological Defects. London: Imperial College Press. doi: 10.1142/p592

[B11] Bogacz R. (2017). A tutorial on the free-energy framework for modelling perception and learning. J. Math. Psychol. 76, 198–211. doi: 10.1016/j.jmp.2015.11.003 28298703 PMC5341759

[B12] Brockman H. (1994). Dipole potential of lipid membranes. Chem. Phys. Lipids 73, 57–79. doi: 10.1016/0009-3084(94)90174-0 8001185

[B13] Bruineberg J. Kiverstein J. Rietveld E. (2018). The anticipating brain is not a scientist. Synthese 195, 2417–2444. doi: 10.1007/s11229-016-1239-1 30996493 PMC6438652

[B14] Buzsáki G. Anastassiou C. A. Koch C. (2012). The origin of extracellular fields and currents. Nat. Rev. Neurosci. 13, 407–420. doi: 10.1038/nrn3241 22595786 PMC4907333

[B15] Calvo P. Friston K. (2017). Predicting green: really radical (plant) predictive processing. J. R. Soc. Interface 14:20170096. doi: 10.1098/rsif.2017.0096 28637913 PMC5493793

[B16] Cavaglià M. Deriu M. A. Tuszynski J. A. (2023). Toward a holographic brain paradigm: a lipid-centric model of brain functioning. Front. Neurosci. 17:1302519. doi: 10.3389/fnins.2023.1302519 38161798 PMC10757614

[B17] Cavaglià M. Tuszynski J. A. (2025a). A unified holographic framework for neural computation and consciousness. BioSystems 259:105669. doi: 10.1016/j.biosystems.2025.105669 41407072

[B18] Cavaglià M. Tuszynski J. A. (2025b). A physical model of neuronal membrane excitations as a mechanism of holographic image formation in the brain. J. Multiscale Neurosci. 4, 187–195. doi: 10.56280/1704135468

[B19] Chiolerio A. Vitiello G. Dehshibi M. M. Crepaldi M. Torazza D. Adamatzky A. . (2026). Observation of phase correlations in ferrofluids. Sci. Rep. 16:16510. doi: 10.21203/rs.3.rs-6813465/v1 41936604 PMC13216242

[B20] Cifra M. Pospíšil P. (2014). Ultra-weak photon emission from biological samples. J. Photochem. Photobiol. B Biol. 139, 2–10. doi: 10.1016/j.jphotobiol.2014.02.009 24726298

[B21] Clark A. (2013). Whatever next? Predictive brains, situated agents, and the future of cognitive science. Behav. Brain Sci. 36, 181–204. doi: 10.1017/S0140525X12000477 23663408

[B22] Clark A. (2015). Surfing Uncertainty. Oxford: Oxford University Press.

[B23] Davies P. Rieper E. Tuszynski J. A. (2012). Self-organization and entropy reduction in a living cell. BioSystems. 111, 1–10. doi: 10.1016/j.biosystems.2012.10.005 23159919 PMC3712629

[B24] Dayan P. Hinton G. E. Neal R. M. Zemel R. S. (1995). The Helmholtz machine. Neural Comput. 7, 889–904. doi: 10.1162/neco.1995.7.5.889 7584891

[B25] Deco G. Kringelbach M. L. Jirsa V. K. Ritter P. (2017). The dynamics of resting fluctuations in the brain. Sci. Rep. 7:3095. doi: 10.1038/s41598-017-03073-5 28596608 PMC5465179

[B26] Del Giudice E. Doglia S. Milani M. Vitiello G. (1986). Electromagnetic field and spontaneous symmetry breaking in biological matter. Nucl. Phys. B 275, 185–199. doi: 10.1016/0550-3213(86)90595-X

[B27] Del Giudice E. Preparata G. Vitiello G. (1988). Water as a free electric dipole laser. Phys. Rev. Lett. 61, 1085–1088. doi: 10.1103/PhysRevLett.61.1085 10039515

[B28] Demetrius L. (1997). Directionality principles in thermodynamics and evolution. Proc. Natl. Acad. Sci. U. S. A. 94, 3491–3498. doi: 10.1073/pnas.94.8.3491 9108003 PMC34158

[B29] Demetrius L. (2013). Boltzmann, Darwin and directionality theory. Phys. Rep. 530, 1–85. doi: 10.1016/j.physrep.2013.04.001

[B30] Demetrius L. A. (2024). Directionality theory and the origin of life. R. Soc. Open Sci. 11, 230623. doi: 10.1098/rsos.230623 39539501 PMC11558456

[B31] Disalvo E. A. Hollmann A. Martini M. F. (2015). Hydration in lipid monolayers. Subcell. Biochem. 71, 213–231. doi: 10.1007/978-3-319-19060-0_9 26438267

[B32] Dreha-Kulaczewski S. Joseph A. A. Merboldt K. D. Ludwig H. C. Gärtner J. Frahm J. (2015). Inspiration is the major regulator of human CSF flow. J. Neurosci. 35, 2485–2491. doi: 10.1523/JNEUROSCI.3246-14.2015 25673843 PMC6605608

[B33] Ebel H. Grabitz P. Heimburg T. (2001). Enthalpy and volume changes in lipid membranes. J. Phys. Chem. B 105, 7353–7360. doi: 10.1021/jp010515s

[B34] Elton D. C. Spencer P. D. Riches J. D. Williams E. D. (2020). Exclusion zone phenomena in water. Int. J. Mol. Sci. 21:5041. doi: 10.3390/ijms21145041 32708867 PMC7404113

[B35] Fields C. Glazebrook J. F. Levin M. (2024). Principled limitations on self-representation for generic physical systems. Entropy 26:194. doi: 10.3390/e26030194 38539706 PMC10969210

[B36] Firaux T. Tuszynski J. A. Cavaglià M. (2025). EMI model: a scale-free biophysical framework for human consciousness, cognition and behaviour. BioSystems 259:105674. doi: 10.1016/j.biosystems.2025.105674 41380798

[B37] Fries P. (2005). A mechanism for cognitive dynamics: neuronal communication through coherence. Trends Cogn. Sci. 9, 474–480. doi: 10.1016/j.tics.2005.08.011 16150631

[B38] Friston K. (2010). The free-energy principle: a unified brain theory? Nat. Rev. Neurosci. 11, 127–138. doi: 10.1038/nrn2787 20068583

[B39] Friston K. (2013). Life as we know it. J. R. Soc. Interface 10:20130475. doi: 10.1098/rsif.2013.0475 23825119 PMC3730701

[B40] Friston K. (2019). A free energy principle for a particular physics. arXiv [Preprint]. *arXiv:1906.10184*. Available online at: https://arxiv.org/abs/1906.10184 (Accessed August 11, 2026).

[B41] Friston K. Levin M. Sengupta B. Pezzulo G. (2015). Knowing one's place: a free-energy approach to pattern regulation. J. R. Soc. Interface 12:20141383. doi: 10.1098/rsif.2014.1383 25788538 PMC4387527

[B42] Friston K. Lin M. Frith C. D. Pezzulo G. Hobson J. A. Ondobaka S. . (2017). Active inference, curiosity and insight. Neural Comput. 29, 2633–2683. doi: 10.1162/neco_a_00999 28777724

[B43] Fröhlich H. (1968). Long-range coherence and energy storage in biological systems. Int. J. Quantum Chem. 2, 641–649. doi: 10.1002/qua.560020505

[B44] Fröhlich H. (1970). Long range coherence and the action of enzymes. Nature 228:1093. doi: 10.1038/2281093a0 5483165

[B45] Fultz N. E. Bonmassar G. Setsompop K. Stickgold R. A. Rosen B. R. Polimeni J. R. . (2019). Coupled electrophysiological, hemodynamic, and cerebrospinal fluid oscillations in human sleep. Science 366, 628–631. doi: 10.1126/science.aax5440 31672896 PMC7309589

[B46] Gabor D. (1948). A new microscopic principle. Nature 161, 777–778. doi: 10.1038/161777a0 18860291

[B47] Gentili P. L. (2025). Chemical AI in the limelight: the contribution of photochromic materials and oscillatory chemical reactions. Adv. Opt. Mater. 13:2500016. doi: 10.1002/adom.202500016

[B48] Gödel K. (1931). Über formal unentscheidbare Sätze der Principia Mathematica und verwandter Systeme I. Monatsh. Math. Phys. 38, 173–198. doi: 10.1007/BF01700692

[B49] Gregory R. L. (1980). Perceptions as hypotheses. Phil. Trans. R. Soc. Lond. B 290, 181–197. doi: 10.1098/rstb.1980.0090 6106237

[B50] Heimburg T. (2023). The excitable fluid mosaic. Biochim. Biophys. Acta Biomembr. 1865:184104. doi: 10.1016/j.bbamem.2022.184104 36642342

[B51] Heimburg T. Jackson A. D. (2005). On soliton propagation in biomembranes and nerves. Proc. Natl. Acad. Sci. U. S. A. 102, 9790–9795. doi: 10.1073/pnas.0503823102 15994235 PMC1175000

[B52] Heimburg T. Jackson A. D. (2007). The thermodynamics of general anesthesia. Biophys. J. 92, 3159–3165. doi: 10.1529/biophysj.106.099754 17293400 PMC1852341

[B53] Hohwy J. (2013). The Predictive Mind. Oxford: Oxford University Press. doi: 10.1093/acprof:oso/9780199682737.001.0001

[B54] Hunt T. Schooler J. W. (2019). The easy part of the hard problem: a resonance theory of consciousness. Front. Hum. Neurosci. 13:378. doi: 10.3389/fnhum.2019.00378 31736728 PMC6834646

[B55] Kageyama H. Komiya M. Yamamoto E. Hirano-Iwata A. (2025). Distinct structural responses of lipid bilayers to horizontal and vertical electric fields. J. Phys. Chem. Lett. 16, 10647**–**10654. doi: 10.1021/acs.jpclett.5c02764 41051904 PMC12536441

[B56] Kanerva P. (2009). Hyperdimensional computing. Cogn. Comput. 1, 139–159. doi: 10.1007/s12559-009-9009-8

[B57] Kirchhoff M. Parr T. Palacios E. Friston K. Kiverstein J. (2018). The Markov blankets of life: autonomy, active inference and the free energy principle. J. R. Soc. Interface 15:20170792. doi: 10.1098/rsif.2017.0792 29343629 PMC5805980

[B58] Knill D. C. Pouget A. (2004). The Bayesian brain: the role of uncertainty in neural coding and computation. Trends Neurosci. 27, 712–719. doi: 10.1016/j.tins.2004.10.007 15541511

[B59] Lakatos P. Karmos G. Mehta A. D. Ulbert I. Schroeder C. E. (2008). Entrainment of neuronal oscillations as a mechanism of attentional selection. Science 320, 110–113. doi: 10.1126/science.1154735 18388295

[B60] Lanillos P. Meo C. Pezzato C. Meera A. A. Baioumy M. Ohata W. . (2021). Active inference in robotics and artificial agents: survey and challenges. arXiv [Preprint]. *arXiv:2112.01871*. Available online at: https://arxiv.org/abs/2112.01871 (Accessed August 11, 2026).

[B61] Lavrentovich M. O. Carrillo J. M. Y. Collier C. P. Katsaras J. Bolmatov D. (2025). Curvature memory in electrically stimulated lipid membranes. Langmuir 41, 3157–3165. doi: 10.1021/acs.langmuir.4c03799 39871541

[B62] Lerchner A. (2026). The Abstraction Fallacy: Why AI Can Simulate but Not Instantiate Consciousness. PhilArchive. Available online at: https://philarchive.org/rec/LERTAF (Accessed August 11, 2026).

[B63] Leszczynski M. Schroeder C. E. (2019). The role of neuronal oscillations in visual active sensing. Front. Integr. Neurosci. 13**:**32. doi: 10.3389/fnint.2019.00032 31396059 PMC6664014

[B64] Levental I. Levental K. R. Heberle F. A. (2020). Lipid rafts: controversies resolved, mysteries remain. Trends Cell Biol. 30, 341–353. doi: 10.1016/j.tcb.2020.01.009 32302547 PMC7798360

[B65] Levin M. (2024). The multiscale wisdom of the body: collective intelligence as a tractable interface for biomedical intervention. Bioessays 46:2300228. doi: 10.31219/osf.io/yq9xe PMC11848127 39623868

[B66] Lingwood D. Simons K. (2010). Lipid rafts as a membrane-organizing principle. Science 327, 46–50. doi: 10.1126/science.1174621 20044567

[B67] Miller W. B. Jr. Baluška F. Torday J S. (2020). Cellular senomic measurements in cognition-based evolution. Prog. Biophys. Mol. Biol. 156, 20–33. doi: 10.1016/j.pbiomolbio.2020.07.002 32738355

[B68] Miller W. B. Jr. Slijepčević P. Baluška F. (2026). Access denied: Plato's cave and the epistemic limits of cellular life. BioSystems 265:105792. doi: 10.1016/j.biosystems.2026.105792 42009120

[B69] Mohamed A. S. Dhungel A. Hasan M. S. Najem J. S. (2024). Intrinsic voltage offsets in memcapacitive biomembranes enable high-performance physical reservoir computing. ACS Appl. Eng. Mater. 2, 2118**–**2130. doi: 10.1021/acsaenm.4c00309

[B70] Nedergaard M. Goldman S. A. (2020). Glymphatic failure as a final common pathway to dementia. Science 370, 50–56. doi: 10.1126/science.abb8739 33004510 PMC8186542

[B71] Nicolis G. Prigogine I. (1977). Self-Organization in Nonequilibrium Systems: From Dissipative Structures to Order Through Fluctuations. New York, NY: Wiley.

[B72] Nishiyama A. Tanaka S. Tuszynski J. A. (2024). Non-equilibrium quantum brain dynamics. Entropy 26:981. doi: 10.3390/e26110981 39593925 PMC11592528

[B73] Palacios E. R. Razi A. Parr T. Kirchhoff M. Friston K. (2020). On Markov blankets and hierarchical self-organisation. J. Theor. Biol. 486:110089. doi: 10.1016/j.jtbi.2019.110089 31756340 PMC7284313

[B74] Parr T. Pezzulo G. Friston K. J. (2022). Active Inference. Cambridge, MA: MIT Press. doi: 10.7551/mitpress/12441.001.0001

[B75] Pearl J. (1988). Probabilistic Reasoning in Intelligent Systems. San Mateo, CA: Morgan Kaufmann.

[B76] Perez-Martin E. Beranger T. Bonnet L. Teppe F. Lisauskas A. Ikamas K. . (2025). Unveiling long-range forces in light-harvesting proteins: pivotal roles of temperature and light. Sci. Adv. 11:eadv0346. doi: 10.1126/sciadv.adv0346 40305607 PMC12042873

[B77] Pio-Lopez L. Kuchling F. Tung A. Pezzulo G. Levin M. (2022). Active inference, morphogenesis, and computational psychiatry. Front. Comput. Neurosci. 16:988977. doi: 10.3389/fncom.2022.988977 36507307 PMC9731232

[B78] Plankar M. BreŽan S. Jerman I. (2013). The principle of coherence in multi-level brain information processing. Prog. Biophys. Mol. Biol. 111, 8–29. doi: 10.1016/j.pbiomolbio.2012.08.006 22986048

[B79] Plate T. A. (1995). Holographic reduced representations. IEEE Trans. Neural Netw. 6, 623–641. doi: 10.1109/72.377968 18263348

[B80] Podar P. T. Bolmatov D. Kumarage T. Ashkar R. Adkisson A. Ziemer O. . (2025). Electromechanically induced membrane restructuring enables learning and memory. Proc. Natl. Acad. Sci. U. S. A. 122:e2510664122. doi: 10.1073/pnas.2510664122 41187087 PMC12625889

[B81] Pollack G. H. (2013). The Fourth Phase of Water. Seattle, WA: Ebner and Sons.

[B82] Purdon P. L. Pierce E. T. Mukamel E. A. Prerau M. J. Walsh J. L. Wong K. F. . (2013). EEG signatures of loss and recovery of consciousness from propofol. Proc. Natl. Acad. Sci. U. S. A. 110, E1142–E1151. doi: 10.1073/pnas.1221180110 23487781 PMC3607036

[B83] Raja V. Valluri D. Baggs E. Chemero A. Anderson M. L. (2021). The Markov blanket trick. On the scope of free energy principle and active inference. Phys. Life Rev. 39, 49–72. doi: 10.1016/j.plrev.2021.09.001 34563472

[B84] Ramstead M. J. D. Albarracin M. Kiefer A. Klein B. Fields C. Friston K. . (2023). The inner screen model of consciousness: applying the free energy principle directly to the study of conscious experience. PsyArXiv [Preprint]. doi: 10.31234/osf.io/6afs3 PMC12013476 40265192

[B85] Ramstead M. J. D. Badcock P. B. Friston K. J. (2018). Answering Schrödinger's question: a free-energy formulation. Phys. Life Rev. 24, 1–16. doi: 10.1016/j.plrev.2017.09.001 29029962 PMC5857288

[B86] Rao R. P. N. Ballard D. H. (1999). Predictive coding in the visual cortex: a functional interpretation of some extra-classical receptive-field effects. Nat. Neurosci. 2, 79–87. doi: 10.1038/4580 10195184

[B87] Renati P. Madl P. (2025). From ‘information' to configuration and meaning: in living systems, the structure is the function. Int. J. Mol. Sci. 26:7319. doi: 10.3390/ijms26157319 40806451 PMC12347946

[B88] Richter C. G. Babo-Rebelo M. Schwartz D. Tallon-Baudry C. (2017). Phase–amplitude coupling at the organism level. Neuroimage 146, 951–958. doi: 10.1016/j.neuroimage.2016.08.043 27557620 PMC5312779

[B89] Rozenberg G. Bäck T. Kok J. N. (eds.). (2012). Handbook of Natural Computing. Berlin: Springer. doi: 10.1007/978-3-540-92910-9

[B90] Sajid N. Ball P. J. Parr T. Friston K. J. (2021). Active inference: demystified and compared. Neural Comput. 33, 674–712. doi: 10.1162/neco_a_01357 33400903

[B91] Samaha J. Postle B. R. (2015). The speed of alpha-band oscillations predicts the temporal resolution of visual perception. Curr. Biol. 25, 2985–2990. doi: 10.1016/j.cub.2015.10.007 26526370 PMC4654641

[B92] Schroeder C. E. Lakatos P. (2009). Low-frequency neuronal oscillations as instruments of sensory selection. Trends Neurosci. 32, 9–18. doi: 10.1016/j.tins.2008.09.012 19012975 PMC2990947

[B93] Seth A. K. (2013). Interoceptive inference, emotion, and the embodied self. Trends Cogn. Sci. 17, 565–573. doi: 10.1016/j.tics.2013.09.007 24126130

[B94] Seth A. K. Friston K. J. (2016). Active interoceptive inference and the emotional brain. Phil. Trans. R. Soc. B 371:20160007. doi: 10.1098/rstb.2016.0007 28080966 PMC5062097

[B95] Shrivastava S. (2018). Detonation of shock-waves and action potentials in lipid interfaces. Proc. Meet. Acoust. 34:045034. doi: 10.1121/2.0000906

[B96] Shrivastava S. (2024). Signal Processing Device with Lipid Interface. US Patent Application 2024/0267135 A1. Alexandria, VA: United States Patent and Trademark Office (USPTO).

[B97] Shrivastava S. Kang K. H. Schneider M. F. (2015). Solitary shock waves and adiabatic phase transition in lipid interfaces and nerves. Phys. Rev. E 91:012715. doi: 10.1103/PhysRevE.91.012715 25679650

[B98] Shrivastava S. Schneider M. F. (2014). Evidence for two-dimensional solitary sound waves in a lipid controlled interface. J. R. Soc. Interface 11:20140098. doi: 10.1098/rsif.2014.0098 24942845 PMC4078894

[B99] Singer S. J. Nicolson G. L. (1972). The fluid mosaic model. Science 175, 720–731. doi: 10.1126/science.175.4023.720 4333397

[B100] Spaak E. de Lange F. P. Jensen O. (2014). Local entrainment of alpha oscillations by visual stimuli causes cyclic modulation of perception. J. Neurosci. 34, 3536–3544. doi: 10.1523/JNEUROSCI.4385-13.2014 24599454 PMC6608988

[B101] Stefanics G. Hangya B. Hernádi I. Winkler I. Lakatos P. Ulbert I. . (2010). Phase entrainment of human delta oscillations can mediate the effects of expectation on reaction speed. J. Neurosci. 30, 13578–13585. doi: 10.1523/JNEUROSCI.0703-10.2010 20943899 PMC4427664

[B102] Tyler A. I. I. Clarke J. A. Seddon J. M. Law R. V. (2015). Solid state NMR of lipid model membranes. Methods Mol. Biol. 1232, 227–253. doi: 10.1007/978-1-4939-1752-5_17 25331139

[B103] Veissière S. P. L. Constant A. Ramstead M. J. D. Friston K. J. Kirmayer L. J. (2020). Thinking through other minds: a variational approach to cognition and culture. Behav. Brain Sci. 43:e90. doi: 10.1017/S0140525X19001213 31142395

[B104] von Helmholtz H. (1867). Handbuch der Physiologischen Optik. Leipzig: Leopold Voss.

[B105] Young A. Hunt T. Ericson M. (2022). The slowest shared resonance: a review of electromagnetic field oscillations between central and peripheral nervous systems. Front. Hum. Neurosci. 15:796455. doi: 10.3389/fnhum.2021.796455 35250508 PMC8888685

[B106] Zizzi E. A. Cavaglià M. Tuszynski J. A. Deriu M. A. (2022). Alteration of lipid bilayer mechanics by volatile anesthetics: insights from μs-long molecular dynamics simulations. iScience 25:103946. doi: 10.1016/j.isci.2022.103946 35265816 PMC8898909

